# Predictors, demographics and frequency of sustained remission and low disease activity in anti-tumour necrosis factor–treated rheumatoid arthritis patients

**DOI:** 10.1093/rheumatology/kez188

**Published:** 2019-06-01

**Authors:** Philip D H Hamann, John D Pauling, Neil McHugh, Gavin Shaddick, Kimme Hyrich, the BSRBR-RA Contributors Group, Nicola Maiden, Tom Price, Neil Hopkinson, Sheila O’Reilly, Lesley Hordon, Ian Griffiths, Duncan Porter, Hilary Capell, Andy Hassell, Romela Benitha, Ernest Choy, David Walsh, Paul Emery, Susan Knight, Ian Bruce, Kimme Hyrich, Allister Taggart, David Scott, Bev Harrison, Paul Thompson, Fiona McCrae, Rhian Goodfellow, Marwan Bukhari, Peter Klimiuk, George Kitas, Ronald Jubb, Rikki Abernethy, Shane Clarke, Sandra Green, Paul Sanders, Amanda Coulson

**Affiliations:** 1 Musculoskeletal Research Unit, University of Bristol, Bristol, UK; 2 Department of Pharmacy and Pharmacology, University of Bath, UK; 3 Royal National Hospital for Rheumatic Diseases, Royal United Hospitals, Bath, UK; 4 College of Engineering, Mathematics and Physical Sciences, University of Exeter, Exeter, UK; 5 Division of Musculoskeletal and Dermatological Sciences, University of Manchester, Manchester, UK

**Keywords:** rheumatoid arthritis, biologic therapies, outcome measures, epidemiology, DMARDS

## Abstract

**Objectives:**

To investigate the frequency and predictors of sustained 28-joint DAS (DAS28) remission and low disease activity (LDA) in patients receiving anti-TNF therapy and changes in responses over a 12 year period.

**Methods:**

Data from the British Society for Rheumatology Biologics Registry for Rheumatoid Arthritis were used. Sustained remission and LDA were defined according to DAS28-ESR thresholds sustained for 6 months. The dataset was dichotomized into sequential chronological subgroups (2001–2010 and 2010–2013). Predictive variables were identified from a previous systematic review and modelled using multivariable logistic regression.

**Results:**

Overall, 2144 (14.9%) and 3802 (26.3%) patients achieved sustained remission or LDA, respectively. Positive predictors of sustained remission/LDA included adalimumab (*vs* etanercept), greater patient global assessment, never- and ex-smoker status (*vs* current smoking), greater swollen joint count, more recent commencement of anti-TNF and MTX co-prescription (except in the 2010–2013 subgroup). Negative predictors of sustained remission and LDA included poor baseline functional status (HAQ), female gender, older age at starting anti-TNF, infliximab use (*vs* etanercept), increasing BMI and greater baseline ESR. Increasing tender joint count was negatively associated with sustained LDA only. The overall proportion of patients achieving sustained remission and LDA has increased significantly over time.

**Conclusion:**

Sustained remission/LDA on anti-TNF treatment remains uncommon. Adalimumab use, greater patient global assessment, never- and ex-smoker status, greater swollen joint count, more recent commencement of anti-TNF and MTX co-prescription are associated with achievement of sustained remission/LDA. However, co-prescription of MTX was not associated with an increased likelihood of achieving sustained remission or LDA in the analysis of more recent anti-TNF responses.

**Rheumatology key messages**

- The demographics of rheumatoid arthritis patients commencing their first anti-TNF has changed significantly since 2001.
- Sustained remission in anti-TNF-treated rheumatoid arthritis patients has increased significantly since 2001.
- Clinical predictors are associated with sustained DAS28 remission in anti-TNF-treated rheumatoid arthritis patients.

## Introduction

Treat-to-target strategies and increased use of biologic agents over the past decade have improved outcomes for patients with RA [1, 2]. Initial use of biologic therapies included patients with long-standing severe disease refractory to conventional synthetic DMARD (csDMARD) therapy. Increasing biologic treatment availability and recognition of the value of treat-to-target strategies have seen them placed earlier in the RA treatment paradigm for patients not responding adequately to csDMARDs [3]. Extensive clinical trial and registry data support the efficacy of anti-TNF biologic drugs [4, 5], although most studies report outcomes at only a single time point. Studies of sustained response to anti-TNF are sparse and evidence suggesting how often and in which patients sustained remission occurs is lacking. Several factors have been identified as being associated with sustained remission in individuals taking both csDMARDs and anti-TNFs [6, 7], although relatively few studies have investigated this outcome. While sustained remission is desirable, low disease activity (LDA) may be an acceptable and more realistic target. Therefore, understanding predictors of sustained LDA is also important.

Anti-TNFs form a routine part of clinical care for patients with RA. Understanding how often and in whom sustained remission/LDA occurs is essential to appreciate the likely success of treatment. With increasing availability and earlier initiation of anti-TNFs and other biologics, it is possible that both the frequency of sustained remission/LDA and predictors associated with these outcomes may have changed over time.

This study investigates the frequency of sustained remission and LDA in patients receiving anti-TNF therapy, the predictors associated with achieving these outcomes and how these predictors and the frequency of these outcomes may have changed over 12 years.

## Methods

The British Society for Rheumatology Biologics Registry for Rheumatoid Arthritis (BSRBR-RA) is a national, prospective, longitudinal, observational study examining the long-term safety of biologic agents in patients with RA in the UK. Ethical approval was obtained from the Multicentre Research Ethics Committee for the North-West of England. All patients enrolled provided written informed consent. The methods of the BSRBR-RA have been described previously [8].

Biologics use in England and Wales is directed by National Institute for Health and Care Excellence guidance [9], which requires patients to have persistent high disease activity [a 28-joint DAS (DAS28) score >5.1 on two occasions, 1 month apart] despite treatment with at least two csDMARDs, one of which should be MTX (unless contraindicated). Therefore the BSRBR-RA is a cohort enriched for high baseline disease activity.

Remission and LDA were defined according to DAS28-ESR thresholds (remission <2.6, LDA 2.6–<3.2). The BSRBR-RA collects DAS28 outcomes on a 6 month basis for the first 3 years. Thereafter, data collection is performed annually. Sustained remission or LDA was defined as any patient achieving the required DAS28-ESR thresholds on two sequential follow-ups during the first 3 years of data collection. Only one period of sustained remission/LDA was counted per individual and analysis included only individuals starting on an anti-TNF as their first biologic agent. Point remission was defined as any patients who had one or more recorded occurrences of non-consecutive episodes of remission. Patient data were censored at the time of switching to another anti-TNF or biologic agent or discontinuation of anti-TNF treatment. Possible clinical and demographic variables associated with response were identified from a systematic review [7] and associations were examined using a generalized linear model, specifically, logistic regression, as the response examined (sustained remission/not sustained remission or LDA/not LDA) was binary.

To examine changes in prescribing and patient characteristics over time, the dataset was split into two chronological subgroups (2001–2010 and 2010–2013). Recruitment for anti-TNF medications (etanercept, infliximab and adalimumab) to the BSRBR-RA was paused from 2007 until 2010 and then restarted in 2010 to include etanercept, infliximab, adalimumab and certolizumab (the recruitment of which was targeted). These factors made 2010 an appropriate time point to split the cohort. Only individuals enrolled up to 2013 were included in the analyses to allow for three complete years of data collection (up to a data censor date of 30 September 2016).

Data were examined to ensure ‘missingness’ occurred at random and bootstrapped multiple imputation was used for missing data. Differences between subgroup baseline characteristics and comparisons of frequency of remission and LDA rates were examined using unpaired *t*-tests and χ^2^. Univariable regression analysis (using a threshold *P*-value of 0.05) confirmed variables for inclusion in the final multivariable logistic regression model. Binary vector multiplication identified individuals in sustained remission/LDA. Uni- and multivariable regression modelling was performed on all imputed datasets [using the generalized linear model package in R (R Foundation for Statistical Computing, Vienna, Austria)] and results combined using Ruben’s rules [10].

## Results

### Changing patient demographics

A total of 14 436 patients with RA starting their first anti-TNF were enrolled between 2001 and 2013. Of these, 13 115 patients were recruited between 2001 and 2010 and 1321 between 2010 and 2013. Due to the BSRBR-RA study design, anti-TNF use was split equally between etanercept, infliximab and adalimumab for the 2001–2010 subgroup. Recruitment targets changed in 2010 and certolizumab was added to the register. In the 2010–2013 subgroup, 659 patients (49.9%) were taking certolizumab, compared with 376 (28.5%), 260 (21.0%) and 26 (2.0%) patients taking etanercept, adalimumab and infliximab, respectively. Baseline MTX use increased over time from 55.9% (7332 patients, 2001–2010 subgroup) to 63.9% (844 patients, 2010–2013 subgroup) (Table 1).

**Table 1:** Change in patient characteristics at the start of the first anti-TNF recorded in the BSRBR-RA over time

Variable	Whole cohort (2001–2013)	2001–2010 subgroup	2010–2013 subgroup	*P*-values^a^ (comparing subgroups)
Patients, *n*	14 436	13 115	1321	NA
Female, %	76.3	76.3	75.7	0.6
Age, mean (s.d.), years	56.0 (12.3)	56.0 (12.2)	56.3 (12.7)	0.4
DAS28-ESR (range 0–10), mean (s.d.)	6.5 (1.0)	6.6 (1.0)	6.0 (1.0)	<0.01
Swollen joint count (range 0–28), mean (s.d.)	11.1 (6.2)	11.4 (6.2)	8.7 (5.2)	<0.01
Tender joint count (range 0–28), mean (s.d.)	15.5 (7.4)	15.6 (7.4)	14.6 (7.5)	<0.01
Patient global assessment (range 0–100 mm), mean (s.d.)	72.5 (19.8)	72.5 (19.8)	72.2 (19.5)	0.6
ESR (mm/h), mean (s.d.)	44.7(28.2)	46.0 (28.3)	29.6 (22.8)	<0.01
HAQ (range 0–3), mean (s.d.)	2.0 (0.6)	2.0 (0.6)	1.6 (0.7)	<0.01
BMI (kg/m^2^), mean (s.d.)	27.2 (8.1)	27.0 (6.8)	29.6 (17.1)	<0.01
Disease duration (years), mean, median (s.d.)	12.7, 11.0 (9.6)	13.0, 11.0 (9.6)	9.6, 6.0 (9.5)	<0.01
Time from first rheumatology consult to biologics (years), mean, median (s.d.)	12.0, 10.0 (9.0)	12.2, 10.0 (8.9)	9.5, 6.0 (9.0)	<0.01
Baseline MTX, *n* (%)	8176 (56.6%)	7332 (55.9%)	844 (63.9%)	<0.01
Etanercept, *n* (%)	4852 (33.6)	4449 (33.9)	376 (28.5)	NA
Infliximab, *n* (%)	4222 (29.2)	4196 (32.0)	26 (2.0)	NA
Certolizumab, *n* (%)	659 (4.6)	0.0	659 (49.9)	NA
Adalimumab, *n* (%)	4730 (32.8)	4471 (34.1)	260 (21.0)	NA
Current smokers, *n* (%)	3108 (21.8)	2861 (22.0)	247 (19.9)	0.03
Ever smoker, *n* (%)	5368 (37.7)	4922 (37.8)	446 (36.0)	
Never smoker, *n* (%)	5778 (40.5)	5232 (40.2)	546 (44.1)	

a Using unpaired *t*-test except gender and smoking data, which used χ^2^.

The mean age, patient global assessment (PGA; measured by visual analogue scale in millimetres) and gender composition at commencement of anti-TNF did not change over time. The median number of swollen joints decreased by nearly three swollen joints, although the number of tender joints only decreased by one. The mean DAS28-ESR, baseline disability (measured by the HAQ), ESR and disease duration (defined as the year of onset of symptoms to the year of commencing anti-TNF) also decreased (*P* < 0.01; Table 1).

BMI in the 2010–2013 subgroup was significantly greater than in the 2001–2010 subgroup. There was a declining trend in current and ex-smokers, with an increasing proportion of never smokers (40.2% to 44.1%, *P* < 0.01). The time from when a patient first saw a rheumatologist to commencement of anti-TNF decreased from a median of 10 to 6 years (*P* < 0.01; Table 1).

### Achievement of sustained remission and LDA

A total of 2144 (14.9%) patients achieved one or more episodes of sustained remission in the whole cohort (Table 2). Point remission was more common [3175 patients (22.0%) in the whole cohort], but still infrequent. The proportion of patients achieving point remission increased between 2001–2010 and 2010–2013 (21.4% *vs* 29.6%, *P* < 0.001; Table 2).

**Table 2:** Frequency of sustained and point remission and LDA over time

Cohort dataset	2001–2013	2001–2010	2010–2013	*P*-value^a^
Patients, *n*	14 436	13 115	1321	NA
Sustained remission, *n* (% of cohort)	2144 (14.9)	1875 (14.3)	285 (21.6)	<0.001
Point remission, *n* (% of cohort)	3175 (22.0)	2802 (21.4)	391 (29.6)	<0.001
Sustained LDA, *n* (% of cohort)				
Any sustained LDA (including sustained remission)	3802 (26.3)	3375 (25.7)	427 (32.3)	<0.001
Sustained LDA (excluding sustained remission) (≥1 episodes point remission ever^b^)	1031 (7.1)	927 (7.1)	106 (8.0)	0.2
Sustained LDA only (no episodes of remission)	627 (4.3)	573 (4.4)	36 (2.7)	0.005

a Unpaired *t*-test.

b Includes patients who may have one or more recorded occurrences of non-consecutive episodes of remission.

Sustained LDA (or better) was also infrequent, with 3802 patients (26.3%) identified in the whole cohort. Of these, 2144 patients (56.4%) were also in sustained remission. Furthermore, the proportion of patients achieving sustained LDA who were also in sustained remission increased from 55.6% (1875 patients, 2001–2010 subgroup) to 66.7% (285 patients, 2010–2013 subgroup; *P* < 0.001).

A large number of patients [1031 (27.1%)] achieving sustained LDA across the whole cohort also had at least one episode of point remission. Indeed, only 627 (16.5%) patients who achieved sustained LDA had no recorded episodes of remission (Table 2).

The proportion of sustained LDA patients achieving at least one episode of point remission has remained stable over time. However, the proportion of patients who never achieved remission in the sustained LDA group decreased from 4.4% in the 2001–2010 subgroup to 2.7% in the 2010–2013 subgroup, although the numbers are small (Table 2).

### Predictors of sustained remission

Univariable analysis (inclusion threshold *P* < 0.05) confirmed the inclusion of all variables in the multivariable model except smoking. However, given the evidence of a link between smoking and RA pathogenesis and severity [11, 12], smoking data were included in the multivariable regression model.

Adalimumab (*vs* etanercept), baseline MTX, greater PGA and ex-smoker status (*vs* current smoker) were associated with an increased likelihood of sustained remission in the whole cohort and the 2001–2010 subgroup. Never-smoker status, greater swollen joint count (SJC) and more recent commencement of anti-TNF were also associated with an improved likelihood of achieving sustained remission in the 2001–2010 subgroup but not the whole cohort. Female gender, older age at starting anti-TNF, infliximab use (*vs* etanercept), increasing BMI and greater baseline ESR were all negatively associated with the likelihood of achieving sustained remission in the cohort as a whole and the 2001–2010 subgroup (Table 3). Poor baseline functional status (HAQ) was the only variable associated with a reduced likelihood of achieving sustained remission in all analyses (Table 3).

**Table 3:** Predictors of sustained remission (multivariable model)

Sustained remission	Whole cohort		2001–2010 subgroup		2010–2013 subgroup	
Variable	OR (95% CI)	*P*-value	OR (95% CI)	*P*-value	OR (95% CI)	*P*-value
Gender (female)	0.59 (0.53, 0.66)	<0.01	0.54 (0.48, 0.60)	<0.01	0.78 (0.56, 1.06)	0.11
HAQ (per unit increase)	0.55 (0.51, 0.60)	<0.01	0.54 (0.50, 0.60)	<0.01	0.57 (0.47, 0.71)	<0.01
DAS28-ESR (per unit increase)	0.92 (0.78, 1.07)	0.27	0.81 (0.68, 0.96)	0.02	1.00 (0.68, 1.45)	0.99
BMI (per kg/m^2^ increase)	0.98 (0.97, 0.99)	<0.01	0.98 (0.97, 0.99)	<0.01	0.98 (0.96, 1.00)	0.10
SWC:TJC (low, moderate, high)	0.99 (0.87, 1.13)	0.89	0.94 (0.82, 1.08)	0.42	1.26 (0.88, 1.81)	0.21
Disease duration (per year increase)	1.00 (1.00, 1.01)	0.83	1.00 (1.00, 1.01)	0.35	0.99 (0.97, 1.00)	0.12
TJC (per unit increase)	0.98 (0.97, 1.00)	0.08	0.99 (0.97, 1.01)	0.25	0.99 (0.95, 1.03)	0.64
SJC (per unit increase)	1.02 (1.00, 1.03)	0.06	1.03 (1.01, 1.04)	<0.01	1.00 (0.95, 1.05)	0.95
PGA (per mm increase)	1.00 (1.00, 1.01)	0.01	1.01 (1.00, 1.01)	<0.01	1.00 (0.99, 1.01)	0.45
ESR (per mm increase)	0.99 (0.98, 0.99)	<0.01	0.99 (0.99, 0.99)	<0.01	0.99 (0.98, 1.00)	0.10
Ex-smoker (*vs* current)	1.16 (1.02, 1.33)	0.02	1.23 (1.06, 1.41)	0.01	0.91 (0.62, 1.32)	0.61
Never smoker (*vs* current)	1.10 (0.97, 1.25)	0.14	1.19 (1.04, 1.37)	0.01	0.84 (0.59, 1.22)	0.36
Age at starting biologic (per year increase)	0.98 (0.98, 0.99)	<0.01	0.98 (0.98, 0.98)	<0.01	1.00 (0.99, 1.01)	0.79
Infliximab (*vs* etanercept)	0.66 (0.57, 0.76)	<0.01	0.66 (0.57, 0.76)	<0.01	0.46 (0.13, 1.60)	0.22
Certolizumab (*vs* etanercept)	–	–	NA^a^	NA^a^	0.91 (0.65, 1.28)	0.59
Adalimumab (*vs* etanercept)	1.29 (1.15, 1.46)	<0.01	1.18 (1.02, 1.36)	0.02	1.16 (0.78, 1.72)	0.46
Year starting anti-TNF (per year)	1.01 (0.99, 1.03)	0.30	1.05 (1.01, 1.09)	0.01	0.98 (0.81, 1.19)	0.82
Baseline MTX	1.48 (1.33, 1.65)	<0.01	1.51 (1.35, 1.70)	<0.01	1.14 (0.85, 1.53)	0.37

a Certolizumab was only licenced for RA after 2010.

### Predictors of sustained LDA

Baseline MTX use, increasing SJC, more recent starting of anti-TNF and ex-smoker status were associated with increased likelihoods of sustained LDA for both the whole cohort and 2001–2010 subgroup and a greater PGA was associated with an increased likelihood of sustained LDA for the 2001–2010 subgroup only. Adalimumab use was associated with an increased likelihood of sustained remission in the whole cohort, but not in the subgroups. A greater SJC:tender joint count (TJC) ratio (i.e. more swollen than tender joints) was associated with a greater chance of achieving sustained LDA in the 2010–2013 subgroup only (Table 4).

**Table 4:** Predictors of sustained LDA

Sustained LDA	Whole cohort		2001–2010 subgroup		2010–2013 subgroup	
Variable	OR (95% CI)	*P*-value	OR (95% CI)	*P*-value	OR (95% CI)	*P*-value
Gender (female)	0.65 (0.60, 0.71)	<0.01	0.63 (0.58, 0.70)	<0.01	0.86 (0.65, 1.15)	0.32
HAQ (per unit increase)	0.61 (0.57, 0.65)	<0.01	0.61 (0.56, 0.65)	<0.01	0.61 (0.51, 0.73)	<0.01
DAS28-ESR (per unit increase)	0.99 (0.86, 1.13)	0.84	0.87 (0.75, 1.01)	0.07	1.18 (0.84, 1.66)	0.33
BMI (per kg/m^2^ increase)	0.98 (0.97, 0.99)	<0.01	0.98 (0.97, 0.99)	<0.01	0.98 (0.96, 1.00)	0.02
SJC:TJC (low, moderate, high)	0.97 (0.87, 1.07)	0.50	0.92 (0.82, 1.02)	0.12	1.46 (1.06, 2.02)	0.02
Disease duration (per year increase)	1.00 (1.00, 1.01)	0.23	1.00 (1.00, 1.01)	0.08	0.99 (0.98, 1.01)	0.41
TJC (per unit increase)	0.97 (0.96, 0.99)	<0.01	0.98 (0.96, 0.99)	0.01	1.00 (0.96, 1.04)	0.90
SJC (per unit increase)	1.01 (1.00, 1.03)	0.07	1.02 (1.01, 1.04)	<0.01	0.98 (0.94, 1.02)	0.38
PGA (per mm increase)	1.00 (1.00, 1.00)	0.21	1.00 (1.00, 1.01)	0.01	1.00 (0.99, 1.01)	0.47
ESR (per mm increase)	0.99 (0.99, 0.99)	<0.01	0.99 (0.99, 0.99)	<0.01	0.99 (0.98, 1.00)	0.01
Ex-smoker (*vs* current)	1.15 (1.03, 1.28)	0.01	1.16 (1.04, 1.30)	0.01	1.08 (0.77, 1.51)	0.68
Never smoker (*vs* current)	1.08 (0.97, 1.20)	0.14	1.11 (0.99, 1.24)	0.08	1.04 (0.75, 1.44)	0.83
Age at starting biologic (per year increase)	0.99 (0.98, 0.99)	<0.01	0.98 (0.98, 0.99)	<0.01	1.00 (0.99, 1.01)	0.56
Infliximab (*vs* etanercept)	0.66 (0.59, 0.73)	<0.01	0.66 (0.59, 0.74)	<0.01	0.21 (0.06, 0.73)	0.01
Certolizumab (*vs* etanercept)	–	–	NA^a^	NA^a^	0.80 (0.59, 1.07)	0.14
Adalimumab (*vs* etanercept)	1.16 (1.05, 1.28)	<0.01	1.06 (0.94, 1.19)	0.33	0.89 (0.63, 1.26)	0.51
Year starting anti-TNF	1.02 (1.00, 1.04)	0.05	1.07 (1.04, 1.11)	<0.01	1.01 (0.85, 1.19)	0.93
Baseline MTX	1.56 (1.43, 1.70)	<0.01	1.57 (1.43, 1.72)	<0.01	1.26 (0.98, 1.63)	0.08

a Certolizumab only licenced for RA after 2010.

Higher HAQ, higher ESR, greater BMI and infliximab (*vs* etanercept) were all associated with reduced likelihoods of sustained LDA in all analyses. Increasing TJC, female gender and greater age at starting anti-TNF treatment were associated with a reduced likelihood of sustained LDA for the cohort as a whole and the 2001–2010 subgroup (Table 4).

## Discussion

This study shows anti-TNF is currently being used in a significantly different patient population compared with when these drugs first became available and much earlier in the disease course. The mean age at anti-TNF commencement has remained constant, most likely due to clinicians prescribing the drug in older patients as well as using it earlier in the disease course. There may also be a changing disease presentation, with later onset related to birth cohort effects [13], ageing populations [14, 15] or changes in environmental factors (e.g. smoking [16]) that may affect RA onset. Although overall disease activity and disability is lower at the start of therapy, the patient perception of the overall disease impact (PGA) has remained static, suggesting the relationship between disease activity and PGA may be non-linear. Psychological and changing health literacy may also play a part, with more accurate symptom reporting using the PGA in the recent subgroup [17]. There may also be a ‘floor effect’ among patients enrolled in the BSRBR-RA due to the minimum DAS28 score of 5.1 required prior to commencing a biologic in the UK.

Outcomes have improved over time, with the proportion of patients achieving either sustained remission or sustained LDA increasing by 7.3% and 6.6%, respectively. This increase is driven by an increasing proportion of patients achieving sustained remission, suggesting clinicians and patients are increasingly successful in targeting remission. Viewed with the data on changing demographics, disease activity, disability and MTX use by patients, these results suggest changes in practice over the past decade are translating into improved outcomes.

The improvements in outcomes over the past 12 years have been accompanied by a change in associations with sustained remission and LDA. A major change is the loss of association between gender and sustained remission/LDA. Female gender was negatively associated with sustained remission in a systematic review [7] and both sustained remission and LDA analyses for the whole cohort and 2001–2010 subgroup analyses (Tables 3 and 4] but not the 2010–2013 subgroup. Possible explanations of this include a relatively small number of men in the 2010–2013 subgroup, unidentified differential selection bias or that men and women have become more similar in reporting RA disease activity. The negative association between BMI and sustained remission/LDA is in keeping with other studies in early [18] and established RA patients taking anti-TNFs [19]. While the statistical significance of the association between BMI and sustained remission is lost in the 2010–2013 subgroup, the direction of the association is the same. It is possible that overall increases in BMI in the 2010–2013 subgroup compared with the 2001–2010 subgroup may have blunted the effect of this relationship.

Although the association between ex-smokers and improved outcomes is in line with existing evidence, no association was identified between never and current smokers. The reason for this is not clear, but may have been influenced by the use of sustained remission/LDA as an outcome rather than the EULAR response criteria that previous studies have used [20, 21]. There may also have been confounding factors related to smoking that influenced this relationship, particularly as smoking was not identified as significant in our initial univariable analyses. Alternatively, it may be that smoking is on the causal pathway of RA. Supporting this, a study of six cohorts identified no association between RA disease severity and smoking when adjusting for ACPA status [22], suggesting poorer outcomes associated with smoking may be mediated by ACPA. ACPA status was not included as a variable in this study, as it has only recently been collected as part of the baseline data in the BSRBR-RA.

The positive association between the SJC and the SJC:TJC ratio with sustained remission/LDA is in keeping with existing evidence [23] and suggests increasing SJCs may be a good clinical predictor of good response to anti-TNF treatment compared with purely elevated TJCs.

In line with current evidence, MTX prescription at baseline showed a strong positive association with sustained remission and LDA. The loss of association between baseline MTX prescription and sustained remission and LDA for the 2010–2013 subgroup could be due to a number of reasons. The most likely explanation is that the 2010–2013 subgroup was insufficiently powered to identify an association, as the direction of the effect in the 2010–2013 subgroup is the same as the overall cohort and 2001–2010 subgroup. Alternatively, this result could be attributed to increasing use of MTX in all patients, changing demographic profiles, different anti-TNF agents used in each subgroup (very little infliximab use in the more recent subgroup), increased anti-TNF switching (and thus being censored from these data) and earlier use of anti-TNFs. However, this study did not investigate switching or length of time on anti-TNF treatment, so it is not possible to quantify this from these data. It should also be noted that for this analysis, MTX use was defined as co-prescription at the time of commencement of an anti-TNF (i.e. baseline entry to the BSRBR-RA), not at subsequent follow-up visits. Therefore it is possible that some patients who were co-prescribed MTX at baseline subsequently stopped taking it and vice versa. Current evidence suggests combination biologic–MTX therapy is superior to biologic monotherapy [24, 25], although this evidence is based on ACR and EULAR responses at one time point rather than sustained remission. This analysis shows that the benefit of co-prescription of MTX with anti-TNF in the 2010–2013 subgroup analysis is less clear and requires further investigation.

Compared with etanercept, adalimumab had better and infliximab worse rates of sustained remission and LDA. Adalimumab may truly be associated with higher rates of sustained remission and LDA. However, etanercept and infliximab were first-in-class anti-TNF agents given to patients with the worst disease activity, greatest disability and longest disease duration, all factors independently associated with a lower likelihood of achieving remission [6, 7] and included in our model. However, a calendar year effect was identified, suggesting additional unmeasured factors have changed over time that may have influenced the results.

In sustained LDA/remission analyses, a greater number of predictors were identified in the 2001–2010 subgroup compared with the 2010–2013 analysis, which may be due to different group sizes, although it is possible increasing treatment standardization has influenced the latter subgroup results.

This analysis examines one of the most challenging clinical targets of treatment: sustained remission. Use of real-world data to examine this endpoint is important, as clinical trial data are lacking and results from routine clinical use are often not as favourable as those seen in clinical trial environments [26]. Furthermore, a clinically pragmatic target of sustained LDA was evaluated. A comprehensive evidence-based range of clinically useful predictors was selected, making these results applicable to clinical settings.

This study has limitations. It is an observational study and, as such, causality cannot be demonstrated. Although recruitment to the BSRBR-RA is nationwide and has broad inclusion criteria, there may be unidentified selection biases. There is real-world variation in follow-up patterns that result in variable sequential follow-up data and there are different recruitment windows that may have influenced results (see Supplementary Table S1, available at *Rheumatology* online). Remission was defined according to DAS28 criteria, which is known to be more lenient than contemporary definitions [27, 28]. The lack of a physician global assessment of disease activity in routine BSRBR-RA data collection precluded analysis of newer composite outcome measures. We have used an evidence-based approach to select the predictors to use in the regression models and other co-morbidities, autoantibodies and radiographic endpoints were not identified as predictors of sustained remission in the previous systematic review [7], so these were not included in this analysis. Furthermore, ACPA status was not included in data collection from registry inception and radiographic outcomes are not collected by the BSRBR-RA.

The sample size of the two subgroups was different, which may have reduced the likelihood of observing associations within the 2010–2013 subgroup. However, this subgroup still included >1300 patients, which was larger than most of the studies identified in our systematic review [7]. We did not stratify analysis by anti-TNF drug, and it is possible that there are variations in predictors of remission/LDA between anti-TNF agents that may have influenced our results (including smoking and MTX). However, further subgroup analysis by anti-TNF drug would have led to small group sizes that would have been underpowered. This analysis did not include data on concomitant DMARDs other than MTX. NSAID or steroid use variables were not included, as the granularity of these data were not sufficient.

### Conclusions

Despite improvement in outcomes, it is sobering to note that between 68% and 78% of patients do not achieve either sustained LDA or remission. 

This study highlights the importance of modifiable factors (reducing BMI and smoking cessation) and is in line with most current standard public health advice. It also shows that clinically demonstrable evidence of inflammation (swollen joints) are a good predictor of future sustained response and supports the paradigm of aggressive treat-to-target clinical practice. Conversely however, it also suggests individuals with high TJCs in the absence of clinical inflammation are less likely to experience resolution of symptoms and is in keeping with findings from a recent Norwegian DMARD study [29].

The finding that MTX does not increase the likelihood of achieving sustained remission or LDA in the most recent subgroup requires further investigation before any changes to guidance can be issued.

These results challenge assumptions about the treatment of RA patients with anti-TNF and show that patients treated in clinical practice today and the associations with sustained remission and LDA are significantly different from when anti-TNF first became available. 

Additional work is required to examine if predictors identified in this study are generic to all biologic-class drugs or if nuances are observed between different biologic classes that could help tailor treatment to individual patients.

## Supplementary Material

kez188_Supplementary_Data
