# Supplementary material for: Predictors, demographics and frequency of sustained remission and low disease activity in anti-tumour necrosis factor–treated rheumatoid arthritis patients
Source: Rheumatology (Oxford). 2019 Jun 1;58(12):2162–9. doi: 10.1093/rheumatology/kez188 (PMC6880851; doi:10.1093/rheumatology/kez188)
Supplement: kez188_Supplementary_Data [file kez188_supplementary_data.docx]

## **SUPPLEMENTARY MATERIAL**

Supplementary Table S1. BSRBR-RA recruitment dates and target cohort sizes.

| **Cohort Details** | **Recruitment Dates** | **Indication** | **Target sample size** |
| --- | --- | --- | --- |
| Enbrel™ (etanercept) | 2001-2005 | RA | 4000 |
| Remicade™ (infliximab) | 2001-2007 | RA | 4000 |
| Humira™ (adalimumab) | 2003-2008 | RA | 4000 |
| Cimzia™ (certlizumab pegol) | 2010 onwards | RA | 2000 |
| Anti-TNF comparator cohort (including infliximab, etanercept and adalimumab) | 2012 onwards | RA | 4000 |

BSRBR-RA: The British Society for Rheumatology Biologics Registry for Rheumatoid Arthritis
